# Supplementary material for: Identification and functional analysis of non-coding regulatory small RNA FenSr3 in Bacillus amyloliquefaciens LPB-18
Source: PeerJ. 2023 May 15;11:e15236. doi: 10.7717/peerj.15236 (PMC10194069; doi:10.7717/peerj.15236)
Supplement: Supplemental Information 4 [file peerj-11-15236-s004.zip › KO/CK-vs-T1_map/map04670.html]

KEGG PATHWAY: Leukocyte transendothelial migration - Reference pathway


|  |  |
| --- | --- |
| **Leukocyte transendothelial migration - Reference pathway** |  |

[
Pathway menu
| Organism menu
| Pathway entry
| Show description
| User data mapping
]

Leukocyte migaration from the blood into tissues is vital for immune surveillance and inflammation. During this diapedesis of leukocytes, the leukocytes bind to endothelial cell adhesion molecules (CAM) and then migrate across the vascular endothelium. A leukocyte adherent to CAMs on the endothelial cells moves forward by leading-edge protrusion and retraction of its tail. In this process, alphaL /beta2 integrin activates through Vav1, RhoA, which subsequently activates the kinase p160ROCK. ROCK activation leads to MLC phosphorylation, resulting in retraction of the actin cytoskeleton. Moreover, Leukocytes activate endothelial cell signals that stimulate endothelial cell retraction during localized dissociation of the endothelial cell junctions. ICAM-1-mediated signals activate an endothelial cell calcium flux and PKC, which are required for ICAM-1 dependent leukocyte migration. VCAM-1 is involved in the opening of the "endothelial passage" through which leukocytes can extravasate. In this regard, VCAM-1 ligation induces NADPH oxidase activation and the production of reactive oxygen species (ROS) in a Rac-mediated manner, with subsequent activation of matrix metallopoteinases and loss of VE-cadherin-mediated adhesion.

|  |  |  |
| --- | --- | --- |
| Reference pathway | 184% 150% 122% 100% 82% 67% 55% | 图片下载 |
